# Characterization and Differentiation of Fresh Orange Juice Variety Based on Conventional Physicochemical Parameters, Flavonoids, and Volatile Compounds Using Chemometrics

**DOI:** 10.3390/molecules27196166

**Published:** 2022-09-20

**Authors:** Maria V. Vavoura, Ioannis K. Karabagias, Ioanna S. Kosma, Anastasia V. Badeka, Michael G. Kontominas

**Affiliations:** 1 Laboratory of Food Chemistry, Department of Chemistry, University of Ioannina, 45110 Ioannina, Greece; 2 Department of Food Science and Technology, School of Agricultural Sciences, University of Patras, 30100 Agrinio, Greece

**Keywords:** orange juice, physico-chemical parameters, flavonoids, volatiles, chemometrics, variety differentiation

## Abstract

The present study focused on the possibility of differentiating fresh-unprocessed orange juice according to botanical origin (variety), based on the use of conventional physico-chemical parameters, flavonoids, and volatile compounds, in combination with chemometrics. For this purpose, oranges from seven different varieties were collected during the harvest years of 2013–2014 and 2014–2015 from central and southern Greece. The physico-chemical parameters that were determined included: electrical conductivity, acidity, pH, and total soluble solids. The flavonoids: hesperidin, neohespseridin, quercetin, naringin, and naringenin were determined using high-performance liquid chromatography (HPLC-DAD). Finally, volatile compounds were determined using headspace solid-phase micro-extraction in combination with gas chromatography-mass spectrometry (HS-SPME/GC-MS). Statistical treatment of data by multivariate techniques showed that orange juice variety had a significant (*p* < 0.05) impact on the above analytical parameters. The classification rate for the differentiation of orange juice according to orange variety using multivariate analysis of variance (MANOVA) and linear discriminant analysis (LDA) was 89.3%, based on the cross-validation method.

## 1. Introduction

The sweet orange (*Citrus sinensis*) is native to China with the orange tree being the most cultivated fruit tree in the world [1]. Oranges have no particular cultivation requirements, although they prefer temperate climates such as those prevailing in the Mediterranean basin. European Union (EU) countries that produce oranges are Spain (50%), and Italy (30%), with the remaining 20% shared by Greece, Portugal, Israel, and Cyprus [2]. The consumption of citrus fruit has been associated with numerous health-promoting benefits including a healthy immune system and the prevention of skin damage, due to their high vitamin C content, control of blood pressure and blood sugar level, promotion of good heart and eye health, the lowering of the risk of colon cancer, lowering of cholesterol, safeguarding against constipation, etc. [3,4]. Likewise, citrus fruit extracts possess antioxidant, anti-inflammatory, anti-tumor, anti-fungal, and blood clot inhibition activity [5].

Oranges are composed of (i) ca. 88% moisture, (ii) ca. 10% sugars (glucose, fructose, and sucrose), (iii) ca. 0.5–1.0% organic acids (citric, malic, and succinic acids), (iv) 0.10–0.13% dietary fiber (in orange juice), made up of mostly pectic substances, (v) ca. 0.1% proteinaceous matter in the form of free amino acids (mainly proline), (vi) antioxidant vitamins C and A, as well as folic acid, (vii) a large number of flavonoids, including: (1) flavanones (hesperidin and naringin), (2) flavones (including flavonols), and (3) anthocyanins, (cyanidin glucoside) of importance in citrus only as red pigments in blood oranges, (viii) phenolic acids (ferrulic acid, hydrocinnamic acid), (ix) carotenoids (β-citraurin in the peel and β- cryptoxanthin in orange juice) and (x) a significant amount of minerals (K, Ca, Fe, Mg, P, and S) [6].

As early as 1992, the EU through the Council Regulations 2081/92 [7] and 2082/92 [8] introduced the terms Protected Designation of Origin (PDO), Protected Geographical Indication (PGI), and Special Traditional Products Guaranteed (STPG), to encourage the production of authentic foodstuffs which add value to respective products. Such foodstuffs are produced using particular raw material, processed in a specific way within a well-defined geographical territory that possesses particular sensory characteristics and specific composition. In 2006, the above regulations were replaced by those of 509/06 [9] and 510/06 [10], however, this came without changing the regulations’ basic scope. Such authentic products enjoy a higher price in domestic and international markets. Therefore, quality and authenticity, including the identification of geographical and botanical origin, are becoming increasingly important for national control organizations. Greece has approved 27 PDO/PGI products among which are fruits, vegetables, and nuts.

Instrumental techniques used for the determination of the botanical and/or geographical origin of foodstuffs include gas chromatography, gas chromatography coupled to mass spectrometry (GC/MS), high-performance liquid chromatography, high-performance liquid chromatography coupled to mass spectrometry (LC/MS), inductively coupled plasma atomic emission spectrometry (ICP-AES), nuclear magnetic resonance spectroscopy (NMR), isotope ratio mass spectroscopy (IRMS), infrared spectroscopy (FTIR), as well as molecular DNA techniques [11,12,13,14,15,16,17,18,19,20,21] in combination with chemometrics.

Based on the above, the objective of the present study was to characterize and differentiate seven orange varieties cultivated in Greece, based on (i) conventional physicochemical parameters, (ii) flavonoids, and (iii) volatile compounds, using chemometrics. The work carried out is the first report in the literature reporting specific qualitative and quantitative data for seven domestically cultivated orange varieties and can be used by either domestic organizations/fruit processors or international authorities, to establish a data bank of orange juice qualitative and quantitative parameters that would aid in product purity control/authenticity.

## 2. Results and Discussion

### 2.1. Conventional Physico-Chemical Parameters of Fresh Orange Juice of Different Varieties

The electrical conductivity of orange juice ranged between 1.67 ± 0.71 mS/cm (samples of the Koino of Rhodes variety) and 2.67 ± 0.48 mS/cm (samples of the Koino of Chios variety). Correspondingly, the total soluble solids ranged from 8.35 ± 3.58 g/100 mL (samples of the Koino of Rhodes variety) to 13.00 ± 3.17 g/100 mL (samples of the Koino of Chios variety). The pH ranged from 3.32 ± 0.09 (samples of the Jaffa variety) to 3.75 ± 0.15 (samples of the Navelina variety). Finally, the titratable acidity ranged from 5.70 ± 0.78 g/L (samples of Valencia variety) to 12.69 ± 4.00 g/L (samples of Koino of Chios variety). The most “acidic” orange juice was that of the Koino of Chios variety, while the least “acidic” was that of the Valencia variety.

It should be noted that three of the four conventional physico-chemical parameters measured, (electrical conductivity, pH, and total soluble solids) showed statistically significant differences (*p* < 0.05) according to the botanical origin of the fresh orange juice (Table 1).

Camin et al. [15] reported pH values between 3.4 and 3.6 for oranges of the Tarocco variety and 3.1–3.7 for the Navelina variety grown conventionally and organically in the Sicily and Calabria regions in Italy. These values are in agreement with the results of the present study, and in particular with the varieties of Koino of Chios, Jaffa, Koino of Rhodes Koino of Arta, and Merlin. The corresponding titratable acidity values were between 12 and 13 g/L for the Tarocco variety and 13 g/L for the Navelina variety. These values are higher than those of the varieties studied in the present work. However, they are in general agreement with the results regarding the variety, Koino of Chios. Differences between the physico-chemical parameter values of Greek vs. Italian Navelina orange juice may be related to soil and microclimate differences between the two countries as well as variety-related differences.

Freshly squeezed oranges of the Navelina variety from different regions of Spain had citric acid levels between 9.52–14.02 g/L [22]. Likewise, Kelebek et al. [12] reported citric acid content in the Turkish Kozan variety to be 12.66 g / L. These values are in good agreement with those for the Koino of the Chios variety. Finally, Nour et al. [14] reported an average citric acid content of 13.92 g/L for orange juice prepared from the sweet variety cultivar (*Citrus sinensis*), cultivated in Romania, which is higher than all acidity values of the present study. It is noteworthy to mention that all the above studies did not report values for electrical conductivity and total dissolved solids. In an effort to differentiate the geographical origin of orange juice belonging to the Merlin variety, Nikolaou et al. [18] reported pH values between 3.3 and 3.7; titratable acidity between 5.73 and 8.79 g/L; electrical conductivity between 1.89 and 2.27 mS/cm and total soluble solids between 9.36–11.4 g/100 mL, values in excellent agreement to those of the present study. Finally, Kim et al. [4] reported values of 3.91 for pH, 17.14 for TSS (Brix = g/100 g), and 2.05% (2.05 g/100 mL) for TA in Navel oranges from Korea. The above pH value is similar to that of the Valencia and Navelina variety of the present study but higher than that of the other orange varieties. The latter is reflected in the higher Brix in the above study compared to that in the present study. With regard to TA, the above value is higher but comparable to those of the present study given that TA in the above study was expressed as percent citric acid in orange fresh weight.

To the best of our knowledge, there is no corresponding study in the literature regarding the differentiation of orange juice according to botanical origin based on the physico-chemical parameters.

### 2.2. Flavonoid Content of Fresh Orange Juice of Different Varieties

Table 2 lists the amounts of flavonoids quantified in the fresh orange juice samples of the seven different varieties. Naringin was not determined in any of the juice samples as it occurs in sour orange varieties [23]. Furthermore, neohesperidin and naringenin were determined in small concentrations (0.74 ± 1.37–5.94 ± 1.01 mg/L and 0.14 ± 0.24–0.27 ± 0.19 mg/L, respectively). Hesperidin and quercetin were the dominant flavonoid compounds in orange juice, determined in all samples. The orange juice samples of the Jaffa variety showed the highest content of hesperidin (435.28 ± 78.62 mg/L) followed by those of Koino of Rhodes (254.87 ± 43.72 mg/L), Koino of Chios (186.83 ± 73.74 mg/L), Navelina (170.58 ± 83.49 mg/L), Valencia (162.35 ± 63.69 mg/L), Koino of Arta (142.86 ± 30.40 mg/L) and Merlin (122.96 ± 31.25 mg/L). Similarly, the orange juice samples with the highest quercetin content were those of the Jaffa variety (17.13 ± 3.33 mg/L), followed by those of Koino of Rhodes (13.17 ± 3.06 mg/L), Koino of Chios (10.54 ± 2.00 mg/L), Navelina (9.99 ± 4.61 mg/L), Koino of Arta (7.51 ± 1.23 mg/L), Merlin (6.85 ± 1.21 mg/L) and Valencia (6.01 ± 2.58 mg/L) varieties. The average flavonoid content of the orange varieties studied in decreasing order was: 459.14 (Jaffa) > 271.32 (Koino of Rhodes) > 197.37 (Koino of Chios) > 180.57 (Navelina) > 168.36 (Valencia) > 151.11 (Koino of Arta) > 129.81 mg/mL (Merlin). In most cases, individual flavonoid concentrations showed statistically significant differences (*p* < 0.05) among orange varieties.

In a similar study dealing with the flavonoid content of six orange varieties (Pera, Natal, Valencia, Hamlin, Baia, and Lima) from Brazil [24], the hesperidin content in orange juice samples varied significantly (*p* < 0.05) according to the botanical origin of the orange, in agreement with the results of the present study. More specifically, the hesperidin concentration range (mg/L) was as follows: Pera 133–399 mg/L, Natal 104–295 mg/L, Valencia 194–321 mg/L, Hamlin 253–537 mg/L, Baia 265–427 mg/L, and Lima 111–223 mg/L. These values are in general agreement with the results of the present study. Belajova and Suhaj [25] studied commercial samples of orange juice from the Slovak region (in Slovakia) and reported values of 2.40–7.00 mg/L for naringin; 44.5–56.10 mg/L for hesperidin; 5.30–11.50 mg/L for neoesperidin and 5.3–7.10 mg/L for quercetin. The hesperidin content values are clearly lower than those of the present study; however, the quercetin content values are in agreement with the present study’s results.

In a more detailed study concerning the flavonoid content of eight orange varieties from the Mediterranean area [26], the hesperidin content varied significantly among varieties. More specifically, the hesperidin content was: 373 mg/L (Salustiana variety), 317 mg/L (Hamlin variety), Maltaise 400 mg/L (Maltaise variety), 552 mg/L (Shamouti variety), 537 mg/L (Sanguinelli variety), 257 mg/L (Valencia variety), 502 mg/L (Pera variety), and 363 mg/L (Cara-cara variety). These values are generally higher than those of the present study. Theodoridis et al. [27] studied the flavonoid content of fresh orange juice from sweet orange varieties and reported naringin content values between 69.41 and 75.36 mg/L. In addition, quercetin was found to be below the detection limit of the method. Finally, Cano et al. [28] studied different Navel varieties (Fukumoto, Lanelate, Navelate, Navelina, Navel Foyos, Newhall and Powell), common variety oranges (Salustiana and Valencia Late), and hematochromes (Sanguinelli) from the region of Valencia (Spain) and reported the following values for hesperidin: Fukumoto: 666 mg/kg, Lane late: 609 mg/kg, Navel late: 648 mg/kg, Navelina: 758 mg/kg, Navel Foyos: 693 mg/kg, Newhall: 1047 mg/kg, and Powell: 747 mg/kg. The average hesperidin content in juice samples of the common orange varieties was: Salustiana: 732 mg/kg and Valencia late: 57.7 mg/kg. Finally, the hesperidin content values in hematochrome orange juice samples were 481 mg/kg. In all cases, the hesperidin content reported by Cano et al. [28] for the orange varieties studied, was higher than the results of the present study. Finally, Proteggente et al. [6] determined, among others, the flavanone content of oranges in Sicily, Italy belonging to the non-pigmented, Ovale, Valencia, and Navel varieties and reported average hesperidin concentrations of 100.75, 52.05, and 121.73 mg/L, respectively. Such hesperidin values are generally lower than those of the present study and similar only to that of the Merlin variety (122.96 mg/L).

To the best of our knowledge, there is very limited data available in the literature [27] reporting the specific flavonoid content of cultivated orange varieties cultivated in Greece, as well as no study available regarding the differentiation of orange juice according to its botanical origin based on flavonoid content.

### 2.3. Semi-Quantitative Data of Volatile Compounds of Fresh Orange Juice of Different Varieties

**Figure 1:**
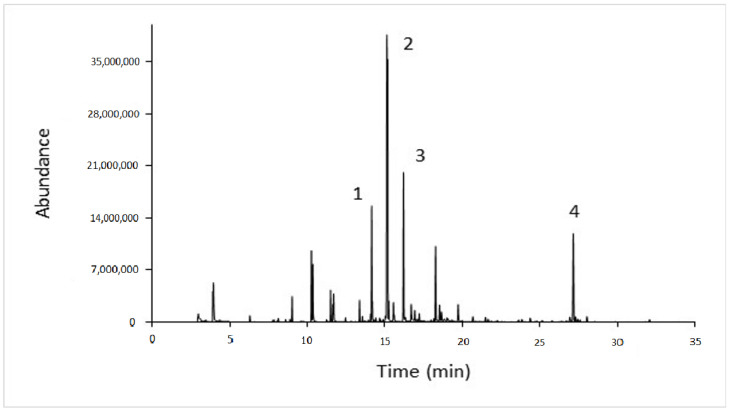
A typical gas chromatogram of fresh orange juice of the Koino Artas cultivar indicating some potential volatile compounds: **1**—beta-myrcene, **2**—dl-limonene, **3**—linalool, **4**—valencene.

Table 3 lists the concentrations of volatile compounds identified in the fresh orange juice of different varieties. Fifty-three volatile compounds were identified and semi-quantified using the internal standard method, belonging to the following six classes: (i) aldehydes, (ii) alcohols, (iii) esters, (iv) ketones, (v) terpenoids, and (vi) hydrocarbons. Figure 1 shows a typical gas-chromatogram of fresh orange juice of the Koino of Arta cultivar, indicating some selected volatile compounds that may be considered as “markers” of the orange juice botanical origin. These volatile compounds have generally been characterized as indicators of the aromatic profile of citrus fruits [29].

Remarkably, terpenoids were the dominant class of aroma compounds of fresh orange juice in all varieties comprising more than 80% of the total amount of orange juice volatile compounds. The most characteristic terpenes that contribute to orange flavor are limonene, pinene, myrcene, phellandrene, delta-3-carene, valencene, sabinene, terpinene, caryophyllene, etc. [30]. dl-limonene was the dominant volatile compound in all orange varieties. Its highest concentration (12.78 ± 5.00 mg/L) was recorded in the orange juice of the Merlin variety and its lowest (4.08 ± 1.84 mg/L) in the Valencia variety. Correspondingly, the volatile compounds linalool, 4-terpineol, valencene, and beta-myrcene were found in varying amounts in the orange juice samples of different varieties and may be considered potential “markers” of the orange botanical origin [31]. Similar variations (*p* < 0.05) in numerous volatile compounds were observed among the different varieties. The volatile compounds p-cymene, hexanal, heptanal, octanol, ethanol, caryophyllene, valencene, alpha-copaene, alpha-terpineol, limonene, decanoic acid ethyl ester, etc. were also found in the Powell Navel Late variety from Valencia [29], in agreement with the results of the present study. Likewise, the volatile compounds sabinene, beta-myrcene, gamma-terpinene, alpha-copaene, *para*-mentha-2,8-dien-1-ol (E), linalool, octanal, cadinene, beta-selinene, carvone, etc. were identified in orange juice samples of the Dortyol variety from eastern Turkey [31], in agreement with present results.

According to Perez-Cacho and Rouseff [30], aldehydes, esters, and terpenoids are considered to be the major constituents of aroma-active compounds in orange juice. Aldehydes are secondary metabolites formed during the plant respiration process and during fruit ripening. They contribute significantly to the aroma of oranges and their concentration increases during the fruit ripening process [31]. Some aldehydes give a fruity aroma to freshly squeezed orange juice, while others give metallic notes. Ethyl esters, such as those identified in the present study, are synthesized from the esterification of ethanol and acyl-CoAs generated from fatty acid and amino acid metabolism [32]. It is noteworthy to mention that of the alcohols identified in the present study, ethanol was the most abundant. According to Perestrelo et al. [21], high ethanol contents were observed mainly in juices without preservatives, particularly freshly-squeezed juices. In general, the formation of flavor in fruits may be the outcome of numerous mechanisms including the lipoxygenase (LOX) pathway and auto-oxidation reactions. A large number of volatile compounds may also be formed in fruits during ripening and handling or processing (mild heat treatment, cutting, chewing, blending, etc.) [20].

In the present work, the volatile compounds identified, showed statistically significant (*p* < 0.05) differences among the different orange varieties (Table 3). The Koino of Chios cultivar possessed the richest aroma (28.70 ± 4.35 mg/L), followed by that of Navelina (19.65 ± 4.79 mg/L), Merlin (18.35 ± 2.59 mg/L), Koino of Arta (18.22 ± 2.60 mg/L), Jaffa (17.63 ± 3.59 mg/L), Koino of Rhodes (16.98 ± 5.63 mg/L) and Valencia (7.76 ± 2.10 mg/L).

In a study by Maccarone et al. [33], 23 volatile compounds were determined in Italian oranges belonging to 4 non-pigmented and 3 blood varieties. The major flavor components (other than limonene) were 2-methyl-1-butanol (0.34 mg/L), valencene (0.21 mg/L), terpinen-4-ol (0.16 mg/L), myrcene (0.15 mg/L), 1-penten-3-ol (0.12 mg/L), linalool (0.083 mg/L), and hexanal (0.075 mg/L). These eight components formed 98% of the aroma in the orange samples studied. All the above compounds, with the exception of 2-methyl-1-butanol, were also identified in the present study. Differences recorded in orange volatiles among studies may be related to differences in orange varieties studied, different soil and climatic conditions prevailing, as well as differences in extraction methodologies employed. Perestrelo et al. [21] determined the volatile compounds of various fresh and processed fruit including oranges. In fresh oranges, they determined 98 volatiles including 41 terpenoids accounting for 53% of volatiles; 23 esters accounting for 23.6% of volatiles; 13 alcohols accounting for 10.1% of volatiles; 16 carbonyl compounds accounting for 9.1% of volatiles; 3 acids, 1 volatile phenol and 1 furanic compound accounting for the remaining 4.2% of volatiles. Finally, Yu et al. [19] reported 84 volatile compounds for blood oranges of the Moro variety of which 37 terpenoids (44.05%), 14 esters (16.67%), 9 aldehydes (10.71%), 7 alcohols (8.33%), 7 ketones (8.33%), 3 hydrocarbons (3.57%) and others (8.33%). Volatile compounds determined in the above studies were in very good agreement with those of the present study.

### 2.4. Differentiation of Fresh Orange Juice According to Variety Based on the Combination of Conventional Physico-Chemical Parameters, Flavonoids, and Volatile Compounds

Application of MANOVA indicated that 58 physico-chemical parameters/flavonoids/volatile compounds were statistically significant variables for the differentiation of the botanical origin of orange juice from 7 different orange varieties. The results showed that six statistically significant functions were formed: The first discriminant function with a canonical correlation of 1.000 and an eigenvalue of 3574,744 accounted for 87.1% of the total variance. Correspondingly, the second discriminant function with a canonical correlation of 0.999 and an eigenvalue of 373,290 accounted for 9.1% of the total variance. Subsequently, the third discriminant function with a canonical correlation of 0.994 and an eigenvalue of 78,882 accounted for 1.9% of the total variance. The fourth discriminant function with a canonical correlation of 0.991 and eigenvalue of 52.091 accounted for 1.3% of the total variance. Similarly, the fifth discriminant function with a canonical correlation of 0.976 and an eigenvalue of 19.951 accounted for 0.5% of the total variance. Finally, the sixth discriminant function with a canonical correlation of 0.918 and an eigenvalue of 5.391 accounted for 0.1% of the total dispersion. All accounted for 100% of the total variance which is an excellent percentage rate.

**Figure 2:**
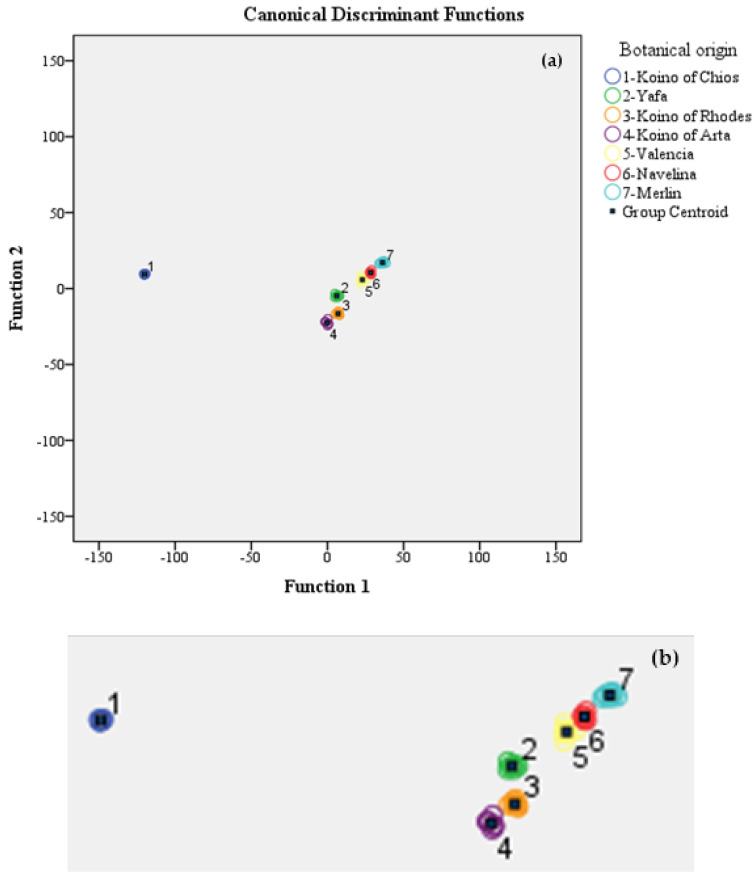
**(a**) Differentiation of fresh orange juice according to variety based on the combination of conventional physico-chemical parameters, flavonoids, and volatile compounds, using LDA. (**b**) Blow up of Figure 2a.

Figure 2 shows the degree of separation of all orange juice varieties based on the cross-validation method. Table 4 lists the correct classification rate of the LDA model based on the original and cross-validation methods. It can be observed that 100% of the samples were classified correctly based on the original method, whereas the correct prediction rate was reduced to 89.3% when using the cross-validation method. This classification rate is very satisfactory, especially for this method of classification. The highest percentage of correct classification was that of the juice samples from the varieties Yaffa and Merlin (100%), followed by those of Koino of Arta, Valencia, and Navelina (87.5%), Koino of Chios (85.7%), and Koino of Rhodes (75%).

In a similar study, Nikolaou et al. [18] attempted to differentiate oranges of the Merlin variety from four different geographical origins in Greece (Messinia, Arta, Rhodes, and Chania-Crete based on organic acids, sugars, and physico-chemical parameters. The authors reported a classification rate of 83.3% based on the combination of organic acids and sugars and 82.0% based on the combination of organic acids, sugars, and physico-chemical parameters using MANOVA and LDA. Maccarone et al. [33] attempted to differentiate 72 Italian orange juice samples belonging to 7 different varieties based on volatile compounds with the aid of LDA. The most important variables for juice differentiation were: myrcene, valencene, terpinen-4-ol, and trans-2-hexenol. These four components were sufficient to correctly classify 66 of 72 juices (91.67%), according to variety. Yu et al. [19] compared juice volatile profiles of 13 selected citrus genotypes, including six mandarins, three sour oranges, one blood orange, one lime, one Clementine, and one Satsuma. Large differences were observed with respect to volatile compositions among the citrus genotypes. Clustering analysis based on the aroma volatile compositions was able to differentiate mandarin varieties and natural sub-groups. No classification rate values, however, were provided in the study. Perestrello et al. [21] achieved the differentiation of both fresh and processed fruit juices, obtained from grapes, red fruits, oranges, pears, and apples by establishing the volatile signature of each using SPME/GC-MS. No classification rate values, however, were provided in the study. According to Gonzalez-Mas et al. [29], the volatile profiling of citrus juices by HS-SPME-GC-MS proved to be a highly valuable tool for the characterization of fruit from different varieties. More specifically using Principal component analysis, the authors successfully differentiated juices from four orange varieties (Powell, Chandler, Clemenules, and Fortune) without providing a specific classification rate. In an effort to distinguish the origin and variety of citrus fruit/fruit juices, Jandric and Cannavan [34] coupled chemometrics and mass spectrometry (UPLC-QToF MS) data. Using partial least squares discriminant analysis (PLS-DA) and soft independent modeling by class analogy (SIMCA), the authors confirmed a 100% recognition ability obtained for hand-squeezed and commercial fresh-squeezed juices. A lower, 80% classification was obtained for commercial orange juice obtained from a concentrate. The discrimination obtained between hand-squeezed and commercial orange juices (fresh-squeezed and prepared from a concentrate) was attributed to changes in the phenolic content caused by the various processing techniques.

Finally, Kim et al. [4] succeeded in categorizing 10 citrus species including 8 mandarin, 1 kumquat and I orange varieties into 4 clusters based on total soluble solids content, titratable acidity, pH, color, total flavonoid content, antioxidant activity, and metabolomic analysis using GC-MS, LC-MS, and HPLC with the aid of PLS-DA and SIMCA chemometric analysis. No specific classification rate was reported in the study.

## 3. Materials and Methods

### 3.1. Orange Samples

Oranges from 7 varieties (Koino of Chios (from Chios island), 8 samples; Jaffa (from Laconia), 8 samples; Koino of Rhodes (from Rhodes island), 8 samples; Koino of Arta (from Arta), 8 samples; Valencia (from Laconia), 8 samples; Navelina (from Laconia), 8 samples and Merlin (from Arta), 8 samples, total 56 samples) were collected from central and southern Greece. Fruits were collected in the harvest years 2013–2014 and 2014–2015 at the commercial ripening stage based on the uniformity of the fruit size and their peel color according to the protocol of the local inspection agronomists. All the orange suppliers, either individual producers or agricultural cooperatives, belong to our laboratory databank created for traceability purposes. Four orange samples (1 kg each) were collected from each orange variety and supplier in the harvest years 2013–14 and 2014–15, respectively. All samples were analyzed in triplicate (*n* = 3) per harvest year.

### 3.2. Preparation of Juice Samples

The oranges were placed in insulated polystyrene boxes and transferred to the Food Chemistry Laboratory within 24 h of their harvesting. Subsequently, the oranges were washed to remove any dirt and other contaminants on the fruit surface and were carefully squeezed in a household juicer. The juice was passed through a strainer to remove pulp and seeds. The strained juice (50 mL) was centrifuged at 3000 rpm for 10 min, and the supernatant was membrane filtered (0.45 μm), collected in polyethylene screw-cap tubes, and stored at −18 °C until further processing for analysis.

### 3.3. Reagents and Solutions

The standard phenolic compounds hesperidin, neohespseridin, quercetin, naringin, and naringenin were purchased from Merck (Darmstadt, Germany). Water, acetonitrile, acetic acid, and sulfuric acid were of HPLC grade and were purchased from Merck (Darmstadt, Germany). The Millex-LCR PTFE filters (0.45 µm) were purchased from Merck (Darmstadt, Germany).

### 3.4. Determination of Conventional Physico-Chemical Parameters

#### 3.4.1. Determination of pH, Electrical Conductivity, Titratable Acidity, and Total Soluble Solids

The pH and electrical conductivity of orange juice samples were measured directly in a 25 mL juice sample using a Delta OHM, model HD 3456.2, pH/conductivity meter (Padova, Italy). Total soluble solids were determined using a model RF6532 refractometer (Euromex, Arnhem, the Netherlands). All measurements were performed in triplicate (*n* = 3) per harvest year at 20 ± 1 °C. Results were expressed as mS/cm for electrical conductivity and (g/100 mL) for TSS.

#### 3.4.2. Determination of Titratable Acidity

Twenty-five mL of orange juice was titrated with sodium hydroxide (0.1 N) using phenolphthalein as the indicator of the end-point. Results were expressed as g citric acid/L of juice [35]. Each sample was analyzed in triplicate (*n* = 3) per harvest year.

### 3.5. Analysis of Flavonoid Compounds

#### 3.5.1. Optimization of the Extraction

Preliminary tests were performed to determine the optimum conditions for juice extraction with respect to the isolation and identification/quantification of flavonoids considering (a) the amount of juice used, (b) the extraction medium, (c) the extraction procedure, and (d) the HPLC gradient elution program.

According to the optimized extraction method, 20 mL of methanol was added to 10 mL of raw, unprocessed juice. The sample was sonicated (Hielscher Ultrasonics, UP200Ht, Teltow, Germany) for 10 min at 10 W and the pulp was removed by filtration. Finally, before HPLC analysis, the free-of-pulp samples were additionally filtered through Millex-LCR PTFE filters (0.45 µm) (Merck, Darmstadt, Germany).

#### 3.5.2. HPLC Instrumentation and Chromatographic Analysis Conditions

The chromatographic analysis of flavonoids was carried out using an HPLC system (Agilent, model 1100 series, Agilent Co., USA). Specific HPLC chromatographic conditions used were determined by trial and error using a mixture of flavonoid standards in an effort to optimize peak resolution, peak height, time of analysis, and the HPLC gradient elution program using different solvents. Gradient elution was used at a flow rate of 1 mL/min using (A) an aqueous solution of 2.0% (*w*/*v*) acetic acid, and (B) acetonitrile of HPLC grade (Merck, Darmstadt, Germany) as the mobile phase. The gradient solvent program was the following: begin with 20% of (B) (0–2 min) then decrease to 10% for 18 min (2–20 min), then increase to 30% for 10 min (20–30 min), further increase to 40% at 31 min (30–31 min), and finally increase to 100% at 40 min (31–40 min). Separation of flavonoids was carried out using a reversed-phase column Eclipse XDB C18 (Merck; 150 mm × 4.5 mm × 5 μm) at room temperature. Hesperidin, neohesperidin, quercetin, naringin, and naringenin were identified at 280 nm. The samples to be analyzed were prepared on the same day and each sample was analyzed in triplicate (*n* = 3).

#### 3.5.3. Qualitative and Quantitative Analysis

**Figure 3:**
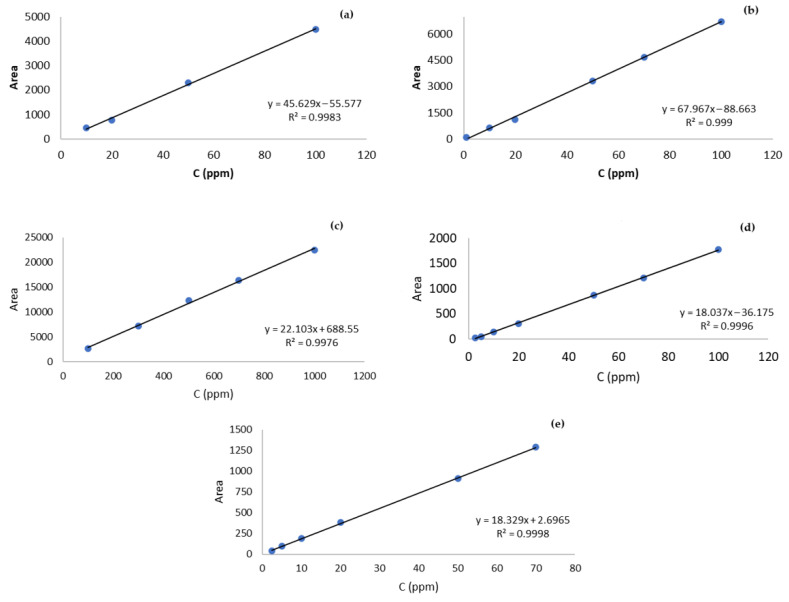
Standard curves of flavonoids: (**a**) quercetin (10–100 ppm), (**b**) naringenin (1–100 ppm), (**c**) hesperidin (100–1000 ppm), (**d**) naringin (2.5–100 ppm), and (**e**) neohesperidin (2.5–70 ppm).

The identification of the studied flavonoids was performed by comparing the retention times of each peak with those of the standard compounds. Quantification of flavonoids was performed by constructing standard curves for different concentrations of each standard compound. The calibration curves were prepared in triplicate for each individual standard at 4–6 different concentrations (1–1000 mg/L). Standard curves are shown in Figure 3.

The determination coefficients for the calibration curves were: R^2^ = 0.9976 for hesperidin, R^2^ = 0.9998 for neohespseridin, R^2^ = 0.9983 for quercetin, R^2^ = 0.9996 for naringin, and R^2^ = 0.999 for naringenin. The limits of detection (LOD) and limit of quantification (LOQ) were: LOD = 1.09 and LOQ = 3.59 mg/L for hesperidin; 2.42 mg/L and 7.98 mg/L for neohespseridin; 2.09 mg/L and 6.88 mg/L for quercetin; 1.51 mg/L and 4.97 mg/L for naringin; and 0.80 and 2.64 for naringenin. The precision and degree of repeatability of the analytical method were tested by calculating the relative standard deviation (RSD%) values of the determined flavonoids. In particular, the respective values were 6.26%, 5.00%, 10.05%, 6.50%, and 6.82% for hesperidin, neohesperidin, quercetin, naringin, and naringenin. Finally, the recovery (%) was calculated on the basis of the difference between the total amount determined in the spiked samples and the amount determined in the non-spiked samples, divided by the amount added. The respective values were 97%, 105%, 75%, 102%, and 93% for hesperidin, neohesperidin, quercetin, naringin, and naringenin.

### 3.6. Semi-Quantitative Determination of Volatile Compounds

Identification and semi-quantitative determination of orange juice volatiles were carried out according to the method of Vavoura [36] using HS-SPME/GC-MS.

Orange juice samples of 5 mL, 1 g NaCl, 30 μL of internal standard 4-methyl-2-pentanone (8 μg/L) (Sigma–Aldrich Co., Munich, Germany) and a microstirring bar were placed in a 20 mL glass vial and sealed with an aluminum crimp-cap equipped with a Teflon-coated needle-pierceable septum supplied by Supelco Co. (St. Louis, MO, USA). Solid-phase microextraction (SPME) was performed with a 75 μm CAR/PDMS fiber (Supelco). The vial was placed in a water bath thermostated at 45 °C and stirred at 700 rpm. After allowing 5 min for the sample to equilibrate, the needle of the SPME device was inserted into the vial through the septum and the fiber was exposed to the headspace of the sample. After 15 min of exposure, the fiber was transferred to the injection port of a gas chromatograph. An Agilent 7890A series gas chromatograph equipped with an Agilent 5975C mass selective detector (USA) was used for the analysis of volatile compounds adsorbed onto the SPME fiber. The column used was a DB-5 MS (60 m × 0.320 mm i.d. and 1 μm film thickness, J & W Scientific, Agilent Technologies, Santa Clara, CA, USA). The flow rate of the helium carrier gas was 1.5 mL/min. The injector temperature was 260 °C in split mode (2:1). The SPME fiber remained in the injector for 10 min. The initial temperature of the column was 40 °C, held for 2 min, heated to 170 °C at a rate of 10 °C/min, then heated to 185 °C at a rate of 2 °C/min followed by heating to 240 °C at a rate 5 °C/min., held for 2 min. MS conditions were as follows: Source temperature: 230 °C; Quadrupole temperature: 150 °C; transfer line temperature: 270 °C; acquisition mode electron impact (EI 70 eV) and mass range m/z: 30–350. Identification of volatile compounds was achieved by comparison of mass spectra of eluting compounds to those of the Wiley library [37]. In addition, the retention index (RI) values of volatile compounds were calculated using the n-alkane (C8–C20) standard (Fluka, Buchs, Switzerland), as well as C5–C7 alkanes dissolved in hexane (Fluka). Semi-quantification of volatiles was achieved by comparing the MS detector response of the internal standard to that of the recorded peaks.

### 3.7. Statistical Analysis

Multivariate analysis of variance (MANOVA) was applied to determine those conventional physico-chemical parameters, flavonoids, and volatile compounds that were significant in differentiating orange juice samples of different botanical origins. The Pillai’s Trace and Wilks’ Lambda indices were computed to determine a possible significant effect of the quantified parameters on the botanical origin of orange juice. LDA was then applied using only the significant dependent variables to explore the possibility of differentiating orange juice samples according to their botanical origin. For the LDA analysis, the botanical origin was taken as the grouping variable (at 7 levels: Koino of Chios, Yafa, Koino of Rhodes, Koino of Arta, Valencia, Navelina, and Merlin), whereas the significant (*p* < 0.05) conventional physico-chemical parameters, flavonoids, and volatile compounds, were taken as the dependent variables. Both the original and leave-one-out cross-validation (LOOCV) (or cross-validation) methods were used to test the prediction ability. The mathematical model that expresses the statistical analysis of LDA in n-selected variables consisting of (i) dividing linear function (F) is of the form:

(1) $$F_{i}=\left(c_{i1}\nu_{1}+c_{i2}\nu_{2}+\ldots {\;c}_{\mathrm{in}}\nu_{n}\right)$$

where v_1_… v_n_ are the values of each variable that was examined and c_i1_, c_i2_,… c_in_ are the correlation coefficients of the differentiation achieved by the above variables [38].

## 4. Conclusions

The results of the present study show that when numerous analytical parameters (i.e., volatile compounds, conventional physico-chemical parameters, and flavonoids) are combined along with chemometric analysis, it is possible to trace the botanical origin of fresh orange juice prepared from seven varieties (correct prediction rate of 89.3%). The study supports the efforts for the preparation of authentic national food products by fruit processors and sets the basis for quality control analysis of different orange varieties in the global market. To the best of our knowledge, there is no similar study in the literature differentiating the orange varieties of Koino of Chios, Yaffa, Koino of Rhodes, Koino of Arta, Valencia, Navelina, and Merlin, this being the major novelty of the study, thus contributing new information to previously published studies regarding fresh/unprocessed orange juice authentication. Furthermore, the present data may aid the fruit juice industry in supplying consumers with premium quality, domestically produced fresh orange juice from specific orange varieties compared to mass imports of orange juice concentrates or elongated shelf-life orange juices. Future research on the subject should concentrate on studies documenting correct differentiation among the above commercial orange juice products through the creation of respective databases.

## Figures and Tables

**Table 1:** Conventional physico-chemical parameters of fresh orange juice of different varieties.

Variety	pH	Titratable Acidity (g/L)	Electrical Conductivity (mS/cm)	Total Dissolved Solids (g/100 mL)
	Average ± SD			
Koino of Chios	3.40 ± 0.10 ^a^	12.69 ± 4.00 ^b^	2.67 ± 0.48 ^b^	13.00 ± 3.17 ^b^
Jaffa	3.32 ± 0.09 ^a^	8.25 ± 1.71 ^a^	1.91 ± 0.29 ^a, b^	9.58 ± 1.43 ^a^^, b^
Koino of Rhodes	3.50 ± 0.83 ^a^	8.55 ± 3.92 ^a^	1.67 ± 0.71 ^a^	8.35 ± 3.58 ^a^
Koino of Arta	3.42 ± 0.22 ^a^	8.14 ± 1.64 ^a^	2.28 ± 0.48 ^a, b^	11.39 ± 2.38 ^a, b^
Valencia	3.72 ± 0.14 ^a^	5.70 ± 0.78 ^a^	2.47 ± 0.58 ^b^	12.07 ± 2.65 ^a, b^
Navelina	3.75 ± 0.15 ^a^	7.03 ± 2.76 ^a^	2.01 ± 0.28 ^a, b^	10.00 ± 1.44 ^a, b^
Merlin	3.40 ± 0.10 ^a^	6.38 ± 2.07 ^a^	1.89 ± 0.61 ^a, b^	9.36 ± 3.01 ^a, b^
*p*	0.086	0.000	0.003	0.011

^a, b, c^ Different letters in each column indicate statistically significant differences at the confidence level *p < 0.05*.

**Table 2:** Flavonoid content of fresh orange juice of different varieties.

Variety	Hesperidin (mg/L)	Neohesperidin (mg/L)	Quercetin (mg/L)	Naringenin (mg/L)
Koino of Chios	186.83 ± 73.74 ^a, b^	nd ^a^	10.54 ± 2.00 ^b, c^	nd ^a^
Jaffa	435.28 ± 78.62 ^c^	5.94 ± 1.01 ^c^	17.13 ± 3.33 ^d^	0.27 ± 0.19 ^b^
Koino of Rhodes	254.87 ± 43.72 ^b^	3.14 ± 2.78 ^b^	13.17 ± 3.06 ^c, d^	0.14 ± 0.24 ^a, b^
Koino of Arta	142.86 ± 30.40 ^a^	0.74 ± 1.37 ^a^	7.51 ± 1.23 ^a, b^	nd ^a^
Valencia	162.35 ± 63.69 ^a, b^	nd ^a^	6.01 ± 2.58 ^a^	nd ^a^
Navelina	170.58 ± 83.49 ^a, b^	nd ^a^	9.99 ± 4.61 ^a, b, c^	nd ^a^
Merlin	122.96 ± 31.25 ^a^	nd ^a^	6.85 ± 1.21 ^a, b^	nd ^a^
*p*	0.000	0.000	0.000	0.000

nd: not determined; ^a, b, c, d^ Different letters in each column indicate statistically significant differences at the confidence level *p* < 0.05.

**Table 3:** Semi-quantification of volatile compounds (mg/L) of fresh orange juice of different varieties.

RT (min)	Volatile Compound	RI_exp_	RI_lit_	Koino of Chios	Jaffa	Koino of Rhodes	Koino of Arta	Valencia	Navelina	Merlin	*p*
				Average ± standard deviation							
	**Aldehydes**										
8.28	Pentanal	706	695	nd ^a^	0.08 ± 0.02 ^c^	0.06 ± 0.03 ^b, c^	0.06 ± 0.02 ^b, c^	nd ^a^	0.05 ± 0.03 ^b, c^	0.04 ± 0.02 ^b^	0.000
10.52	Hexanal	809	804	0.38 ± 0.10 ^a, b^	0.91 ± 0.25 ^c^	0.53 ± 0.22 ^a, b^	0.53 ± 0.20 ^a, b^	0.28 ± 0.23 ^a^	0.64 ± 0.31 ^b, c^	0.39 ± 0.16 ^a, b^	0.000
12.62	Heptanal	911	903	0.05 ± 0.02 ^a^	0.11 ± 0.02 ^a^	0.05 ± 0.03 ^a^	0.07 ± 0.02 ^a^	0.04 ± 0.02 ^a^	0.11 ± 0.11 ^a^	0.09 ± 0.04 ^a^	0.018
14.57	Octanal	1014	1005	0.55 ± 0.58 ^b^	nd ^a^	nd ^a^	nd ^a^	nd ^a^	0.42 ± 0.57 ^a, b^	0.05 ± 0.01 ^b^	0.001
16.32	Nonanal	1109	1106	0.16 ± 0.18 ^b^	nd ^a^	nd ^a^	nd ^a^	nd ^a^	nd ^a^	nd ^a^	0.000
18.58	Decanal	1217	1206	0.39 ± 0.41 ^b^	nd ^a^	nd ^a^	nd ^a^	nd ^a^	nd ^a^	nd ^a^	0.000
	**Total**			**1.53 ± 0.74**	**1.10 ± 0.25**	**0.64 ± 0.22**	**0.66 ± 0.20**	**0.32 ± 0.23**	**1.22 ± 0.66**	**0.57 ± 0.17**	
	**Alcohols**										
4.05	Ethanol	<600	<600	1.73 ± 0.32 ^c^	0.98 ± 0.39 ^a, b^	0.64 ± 0.33 ^a, b^	1.02 ± 0.29 ^b^	0.47 ± 0.30 ^a^	0.90 ± 0.47 ^a, b^	0.63 ± 0.33 ^a, b^	0.000
7.81	1-Penten-3-ol	682	686	nd ^a^	0.02 ± 0.00 ^b^	Nd ^a^	nd ^a^	nd ^a^	nd ^a^	nd ^a^	0.000
11.70	3-Hexen-1-ol (Z)	864	855	0.15 ± 0.09 ^a^	0.46 ± 0.20 ^b^	0.31 ± 0.27 ^a, b^	0.26 ± 0.11 ^a, b^	0.14 ± 0.06 ^a^	0.20 ± 0.12 ^a^	0.14 ± 0.11 ^a^	0.001
11.82	2-Hexen-1-ol (E)	872	872	0.09 ± 0.06 ^b^	0.30 ± 0.21 ^b^	0.07 ± 0.08 ^b^	0.06 ± 0.06 ^b^	nd ^a^	0.10 ± 0.06 ^b^	0.05 ± 0.02 ^b^	0.000
11.87	Hexanol	874	876	0.12 ± 0.08 ^a^	0.36 ± 0.21 ^b^	0.16 ± 0.18 ^a^	0.18 ± 0.09 ^a, b^	0.22 ± 0.11 ^a, b^	0.18 ± 0.07 ^a, b^	0.09 ± 0.05 ^a^	0.002
15.73	Octanol	1077	1070	nd ^a^	nd ^a^	0.18 ± 0.22 ^a, b^	0.19 ± 0.13 ^a, b^	0.18 ± 0.12 ^a, b^	0.33 ± 0.45 ^b^	0.13 ± 0.04 ^a, b^	0.026
	**Total**			**2.09 ± 0.06**	**2.12 ± 0.53**	**1.36 ± 0.52**	**1.71 ± 0.35**	**1.01 ± 0.35**	**1.71 ± 0.67**	**0.99 ± 0.35**	
	**Esters**										
6.45	Acetic acid ethyl ester	613	612	0.24 ± 0.08 ^c^	0.08 ± 0.04 ^b^	nd ^a^	nd ^a^	nd ^a^	nd ^a^	nd ^a^	0.000
10.42	Butanoic acid ethyl ester	805	802	1.07 ± 0.41 ^c^	0.57 ± 0.43 ^b^	0.09 ± 0.09 ^a^	0.34 ± 0.24 ^a, b^	0.18 ± 0.20 ^a, b^	0.31 ± 0.12 ^a, b^	0.45 ± 0.18 ^a, b^	0.000
11.27	2-Butenoic acid ethyl ester	845	844	0.05 ± 0.02 ^b^	nd ^a^	nd ^a^	nd ^a^	nd^a^	nd ^a^	nd ^a^	0.000
14.22	Hexanoic acid ethyl ester	996	996	nd ^a^	nd ^a^	nd ^a^	nd ^a^	0.04 ± 0.07	nd ^a^	nd ^a^	0.029
14.51	2-Hexen-1-ol acetic acid ester	1011	1016	nd ^a^	0.07 ± 0.07 ^b^	nd^a^	nd^a^	nd ^a^	nd ^a^	nd ^a^	0.000
16.89	3-Hydroxy- hexanoic acid ethyl ester	1137	1128	nd ^a^	nd ^a^	nd ^a^	0.12 ± 0.11 ^b^	nd ^a^	nd ^a^	0.11 ± 0.06 ^b^	0.000
18.17	Octanoic acid ethyl ester	1200	1198	0.10 ± 0.03 ^c^	nd ^a^	nd ^a^	0.05 ± 0.02 ^b^	nd ^a^	nd ^a^	0.05 ± 0.01 ^b^	0.000
23.19	Decanoic acid ethyl ester	1392	1393	0.05 ± 0.02 ^b^	nd ^a^	nd ^a^	nd ^a^	nd ^a^	nd ^a^	nd ^a^	0.000
	**Total**			**1.51 ± 0.42**	**0.72 ± 0.44**	**0.09 ± 0.09**	**0.46 ± 0.26**	**0.22 ± 0.21**	**0.31 ± 0.12**	**0.61 ± 0.19**	
	**Ketones**										
7.89	2-Pentanone	687	686	nd ^a^	0.03 ± 0.01 ^b^	nd ^a^	nd ^a^	nd ^a^	nd ^a^	nd ^a^	0.000
	**Terpenoids**										
13.54	alpha-Pinene	959	936	0.44 ± 0.28 ^b^	0.31 ± 0.18 ^a, b^	0.41 ± 0.36 ^a, b^	0.31 ± 0.14 ^a, b^	0.10 ± 0.07 ^a^	0.34 ± 0.23 ^a, b^	0.14 ± 0.05 ^a, b^	0.020
14.32	beta-Myrcene	1000	986	2.10 ± 1.01 ^b^	1.59 ± 0.78 ^a, b^	1.95 ± 1.31 ^b^	1.76 ± 0.58 ^a, b^	0.57 ± 0.43 ^a^	1.58 ± 0.77 ^a, b^	1.02 ± 0.33 ^a, b^	0.006
14.33	beta-Pinene	1001	989	0.08 ± 0.04 ^c^	nd ^a^	nd ^a^	nd ^a^	nd ^a^	0.04 ± 0.02 ^b^	nd ^a^	0.000
14.88	Phelandrene	1031	1010	0.10 ± 0.06 ^b^	nd ^a^	nd ^a^	nd ^a^	nd ^a^	nd ^a^	nd ^a^	0.000
14.94	delta-3-Carene	1034	1020	0.05 ± 0.06 ^b^	nd ^a^	nd ^a^	nd ^a^	nd ^a^	0.24 ± 0.22 ^c^	nd ^a^	0.000
15.07	alpha-Terpinene	1041	1023	0.11 ± 0.06 ^c^	nd ^a^	nd ^a^	nd ^a^	nd ^a^	0.05 ± 0.04 ^b^	nd ^a^	0.000
15.19	p-Cymene	1049	1034	0.68 ± 0.55 ^b^	0.09 ± 0.02 ^a^	nd ^a^	nd ^a^	nd ^a^	nd ^a^	nd ^a^	0.000
15.29	dL-Limonene	1055	1039	11.66 ± 3.84 ^b^	8.11 ± 3.21 ^a, b^	8.93 ± 5.27 ^a, b^	8.30 ± 1.94 ^a, b^	4.08 ± 1.84 ^a^	10.65 ± 4.30 ^b^	12.78 ± 5.00 ^b^	0.001
15.44	Sabinene	1062	-	0.51 ± 0.26 ^b^	0.32 ± 0.21 ^a, b^	0.45 ± 0.34 ^a, b^	0.38 ± 0.14 ^a, b^	0.14 ± 0.10 ^a^	0.40 ± 0.27 ^a, b^	0.18 ± 0.09 ^a, b^	0.013
15.77	gamma-Terpinene	1080	1065	0.91 ± 0.85 ^b^	0.10 ± 0.08 ^a^	0.14 ± 0.11 ^a^	0.13 ± 0.06 ^a^	nd ^a^	0.15 ± 0.11 ^a^	nd ^a^	0.000
16.39	Linalool	1112	1103	2.31 ± 2.14 ^b^	0.68 ± 0.53 ^a^	1.61 ± 1.11 ^a, b^	1.31 ± 0.40 ^a, b^	0.61 ± 0.56 ^a^	1.06 ± 1.01 ^a, b^	0.42 ± 0.13 ^a^	0.009
17.42	(Z)-p-Mentha-2,8-dien-1-ol	1163	1149	nd ^a^	0.09 ± 0.03 ^c^	0.08 ± 0.06 ^c^	0.09 ± 0.03 ^c^	0.04 ± 0.02 ^a, b^	nd ^a^	0.06 ± 0.01 ^b, c^	0.000
18.48	4-Terpineol	1213	1185	1.71 ± 0.80 ^b^	0.47 ± 0.27 ^a^	0.55 ± 0.41 ^a^	0.79 ± 0.32 ^a^	0.25 ± 0.32 ^a^	0.74 ± 0.68 ^a^	0.28 ± 0.12 ^a^	0.000
18.56	beta-Phenyl-alcohol	1216	-	nd ^a^	0.09 ± 0.05 ^b^	nd ^a^	0.27 ± 0.36 ^b, c^	nd ^a^	nd ^a^	nd ^a^	0.001
18.74	alpha-Terpineol	1224	1191	0.82 ± 0.77 ^b^	nd ^a^	0.16 ± 0.11	nd ^a^	0.07 ± 0.06 ^b^	0.37 ± 0.49 ^a, b^	nd ^a^	0.000
18.88	dihydro-Carvone	1230	-	nd ^a^	0.07 ± 0.03 ^b^	nd ^a^	nd ^a^	nd ^a^	nd ^a^	nd ^a^	0.000
19.05	(E)-p-Mentha-6,8-dien-2-ol	1237	1243	0.08 ± 0.08 ^b^	nd ^a^	0.06 ± 0.03 ^b^	nd ^a^	nd ^a^	nd ^a^	nd ^a^	0.000
19.95	delta-Carvone	1275	1243	0.14 ± 0.06 ^a, b^	0.37 ± 0.20 ^c^	0.27 ± 0.15 ^a, b, c^	0.31 ± 0.14 ^b, c^	0.09 ± 0.09 ^a^	0.22 ± 0.12 ^a, b, c^	0.22 ± 0.06 ^a, b, c^	0.001
20.92	Perillaldehyde	1313	1309	0.09 ± 0.05 ^a, b^	0.07 ± 0.04 ^a, b^	0.19 ± 0.30 ^b^	0.08 ± 0.04 ^a, b^	nd ^a^	nd ^a^	0.03 ± 0.01 ^a, b^	0.031
23.89	alpha-Copaene	1416	1390	0.08 ± 0.05 ^b, c^	nd ^a^	nd ^a^	0.13 ± 0.11 ^c^	nd ^a^	0.04 ± 0.02 ^b^	0.02 ± 0.02 ^a, b^	0.000
24.09	beta-Elemene	1423	1445	0.10 ± 0.06 ^b^	0.07 ± 0.08 ^b^	nd ^a^	nd ^a^	nd ^a^	0.05 ± 0.04 ^b^	nd ^a^	0.000
25.41	Caryophyllene	1468	1451	0.08 ± 0.03 ^c^	nd ^a^	nd ^a^	nd ^a^	nd ^a^	0.05 ± 0.03 ^b^	nd ^a^	0.000
27.19	β-Selinene	1530	1490	0.20 ± 0.07 ^c^	nd ^a^	nd ^a^	nd ^a^	0.04 ± 0.03 ^b^	0.11 ± 0.08 ^b^	nd ^a^	0.000
27.20	γ-Selinene	1533	-	nd ^a^	nd ^a^	nd ^a^	nd ^a^	nd ^a^	nd ^a^	0.07 ± 0.05 ^b^	0.000
27.41	Valencene	1538	1493	2.49 ± 0.85 ^c^	2.08 ± 0.98 ^c^	0.55 ± 0.37 ^a, b^	1.78 ± 1.39 ^b, c^	0.39 ± 0.40 ^a^	1.36 ± 1.00 ^a, b, c^	1.36 ± 0.57 ^a, b, c^	0.000
27.55	α-Selinene	1543	1505	0.17 ± 0.06 ^b^	0.13 ± 0.06 ^a, b^	0.05 ± 0.03 ^a^	0.13 ± 0.11 ^a, b^	0.03 ± 0.03 ^a^	0.09 ± 0.07 ^a, b^	0.07 ± 0.04 ^a^	0.001
27.70	Ledene	1548	1511	0.04 ± 0.03 ^b^	nd ^a^	nd ^a^	nd ^a^	nd ^a^	nd ^a^	nd ^a^	0.000
27.84	delta-Cadinene	1553	1536	0.06 ± 0.04 ^b^	nd ^a^	0.09 ± 0.10 ^c^	0.10 ± 0.09 ^c^	nd ^a^	nd ^a^	0.03 ± 0.02 ^b^	0.000
28.29	α-Panasinsene	1569	1577	0.16 ± 0.06 ^c^	0.12 ± 0.06 ^b, c^	0.04 ± 0.02 ^a^	0.13 ± 0.12 ^b, c^	0.03 ± 0.02 ^a^	0.09 ± 0.07 ^a, b^	0.07 ± 0.04 ^a, b^	0.001
	**Total**			**25.10 ± 4.21**	**14.76 ± 3.52**	**15.53 ± 5.61**	**16.00 ± 2.56**	**6.44 ± 2.04**	**17.63 ± 44.69**	**16.75 ± 5.05**	
	**Hydrocarbons**										
8.16	Heptane	700	700	nd ^a^	nd ^a^	nd ^a^	nd ^a^	0.09 ± 0.04 ^b^	nd ^a^	nd ^a^	0.000
16.46	(E) 4,8-dimethyl -1,3,7-nonatriene	1116	1118	nd ^a^	nd ^a^	nd ^a^	0.05 ± 0.03 ^b^	nd ^a^	nd ^a^	nd ^a^	0.000
	**Sum of volatiles**			**28.70 ± 4.35**	**17.63 ± 3.59**	**16.98 ± 5.63**	**18.22 ± 2.60**	**7.76 ± 2.10**	**19.65 ± 4.79**	**18.35 ± 2.59**	

RT: retention time. RI_exp_: experimental retention indices values based on the calculations using the standard mixture of alkanes. RI_lit_: Retention indices of the identified compounds according to the literature data cited in Wiley 7 NIST MS library. nd: not determined. ^a, b, c^ Different letters in each row indicate statistically significant (*p* < 0.05) differences.

**Table 4:** Differentiation ability of the LDA model based on conventional physico-chemical parameters, flavonoids and volatile compounds, for 7 different varieties of fresh orange juice.

Chemometric Technique	Differentiation Rate	Botanical Origin	Predicted Group Membership							Orange Juice Samples
LDA	%		Koino of Chios	Yaffa	Koino of Rhodes	Koino of Arta	Valencia	Navelina	Merlin	
Original	Count	Koino of Chios	8	0	0	0	0	0	0	8
		Yaffa	0	8	0	0	0	0	0	8
		Koino of Rhodes	0	0	8	0	0	0	0	8
		Koino of Arta	0	0	0	8	0	0	0	8
		Valencia	0	0	0	0	8	0	0	8
		Navelina	0	0	0	0	0	8	0	8
		Merlin	0	0	0	0	0	0	8	8
	%	Koino of Chios	100.0	0.0	0.0	0.0	0.0	0.0	0.0	100.0
		Yaffa	0.0	100.0	0.0	0.0	0.0	0.0	0.0	100.0
		Koino of Rhodes	0.0	0.0	100.0	0.0	0.0	0.0	0.0	100.0
		Koino of Arta	0.0	0.0	0.0	100.0	0.0	0.0	0.0	100.0
		Valencia	0.0	0.0	0.0	0.0	100.0	0.0	0.0	100.0
		Navelina	0.0	0.0	0.0	0.0	0.0	100.0	0.0	100.0
		Merlin	0.0	0.0	0.0	0.0	0.0	0.0	100.0	100.0
Cross-validated	Count	Koino of Chios	7	1	0	0	0	0	0	8
		Yaffa	0	8	0	0	0	0	0	8
		Koino of Rhodes	0	0	6	2	0	0	0	8
		Koino of Arta	0	0	1	7	0	0	0	8
		Valencia	0	0	1	0	7	0	0	8
		Navelina	0	0	0	0	0	7	1	8
		Merlin	0	0	0	0	0	0	8	8
	%	Koino of Chios	85.7	14.3	0.0	0.0	0.0	0.0	0.0	100.0
		Yaffa	0.0	100.0	0.0	0.0	0.0	0.0	0.0	100.0
		Koino of Rhodes	0.0	0.0	75.0	25.0	0.0	0.0	0.0	100.0
		Koino of Arta	0.0	0.0	12.5	87.5	0.0	0.0	0.0	100.0
		Valencia	0.0	0.0	12.5	0.0	87.5	0.0	0.0	100.0
		Navelina	0.0	0.0	0.0	0.0	0.0	87.5	12.5	100.0
		Merlin	0.0	0.0	0.0	0.0	0.0	0.0	100.0	100.0

## Data Availability

Data supporting reported results are available from the authors.

## Sample Availability

Samples of the compounds used in the study are available from the authors.
